# Stretching Skeletal Muscle: Chronic Muscle Lengthening through Sarcomerogenesis

**DOI:** 10.1371/journal.pone.0045661

**Published:** 2012-10-01

**Authors:** Alexander M. Zöllner, Oscar J. Abilez, Markus Böl, Ellen Kuhl

**Affiliations:** 1 Department of Mechanical Engineering, Stanford University, Stanford, California, United States of America; 2 Department of Surgery, Stanford University, Stanford, California, United States of America; 3 Department of Mechanical Engineering, TU Braunschweig, Braunschweig, Germany; 4 Departments of Mechanical Engineering, Bioengineering, and Cardiothoracic Surgery, Stanford University, Stanford, California, United States of America; University of California at Davis, United States of America

## Abstract

Skeletal muscle responds to passive overstretch through sarcomerogenesis, the creation and serial deposition of new sarcomere units. Sarcomerogenesis is critical to muscle function: It gradually re-positions the muscle back into its optimal operating regime. Animal models of immobilization, limb lengthening, and tendon transfer have provided significant insight into muscle adaptation in vivo. Yet, to date, there is no mathematical model that allows us to predict how skeletal muscle adapts to mechanical stretch in silico. Here we propose a novel mechanistic model for chronic longitudinal muscle growth in response to passive mechanical stretch. We characterize growth through a single scalar-valued internal variable, the serial sarcomere number. Sarcomerogenesis, the evolution of this variable, is driven by the elastic mechanical stretch. To analyze realistic three-dimensional muscle geometries, we embed our model into a nonlinear finite element framework. In a chronic limb lengthening study with a muscle stretch of 1.14, the model predicts an acute sarcomere lengthening from 3.09
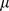
m to 3.51
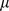
m, and a chronic gradual return to the initial sarcomere length within two weeks. Compared to the experiment, the acute model error was 0.00% by design of the model; the chronic model error was 2.13%, which lies within the rage of the experimental standard deviation. Our model explains, from a mechanistic point of view, why gradual multi-step muscle lengthening is less invasive than single-step lengthening. It also explains regional variations in sarcomere length, shorter close to and longer away from the muscle-tendon interface. Once calibrated with a richer data set, our model may help surgeons to prevent muscle overstretch and make informed decisions about optimal stretch increments, stretch timing, and stretch amplitudes. We anticipate our study to open new avenues in orthopedic and reconstructive surgery and enhance treatment for patients with ill proportioned limbs, tendon lengthening, tendon transfer, tendon tear, and chronically retracted muscles.

## Introduction

Striated muscle displays the striking ability to rapidly adapt to changes in physiological requirements through the dynamic assembly and disassembly of its functional building blocks, the sarcomeres [1]. Sarcomeres are characterized through a parallel arrangement of thick filaments of myosin that slide along thin filaments of actin [2].

**Figure 1:**
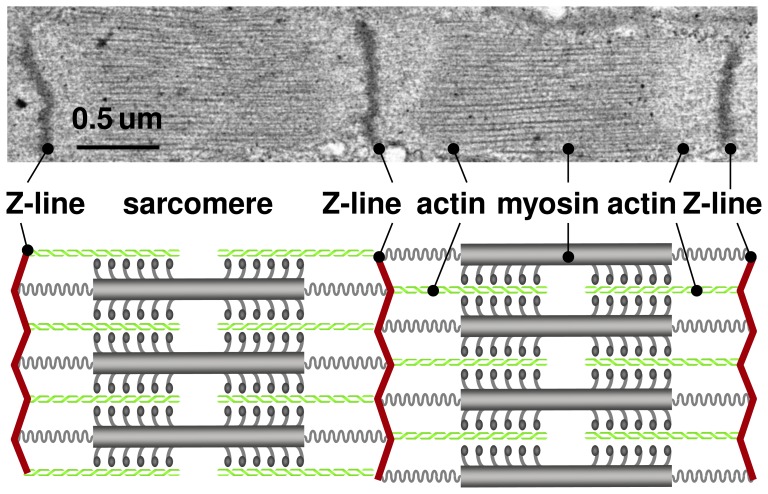
Sarcomere units in striated muscle. Sarcomeres consist of a parallel arrangement of thick filaments of myosin (gray) sliding along thin filaments of actin (green). They are embedded between Z-lines (red), which appear as dark lines under the transmission electron microscope. In healthy muscle, through the dynamic assembly and disassembly, individual sarcomere units maintain an optimal operating length. Adopted with permission from [16].

Figure 1 illustrates two sarcomere units embedded between neighboring Z-lines. Under the transmission electron microscope, Z-lines appear as dark lines giving the muscle its characteristic striated appearance [3]. The appropriate overlap of actin and myosin filaments is critical to active muscle contraction, and sarcomeres produce their maximum force at a characteristic optimal sarcomere length [4]. When stretched beyond the physiological limit, skeletal muscles responds through sarcomerogenesis [5], the creation and serial deposition of new sarcomere units [6], to gradually return into its optimal operating regime [7]. This dynamic adjustment is key to long-term regeneration and repair durability in orthopedic and reconstructive surgery. Typical examples are surgical limb lengthening, tendon lengthening, tendon transfer, or tendon reattachment after tendon tear.

**Figure 2:**
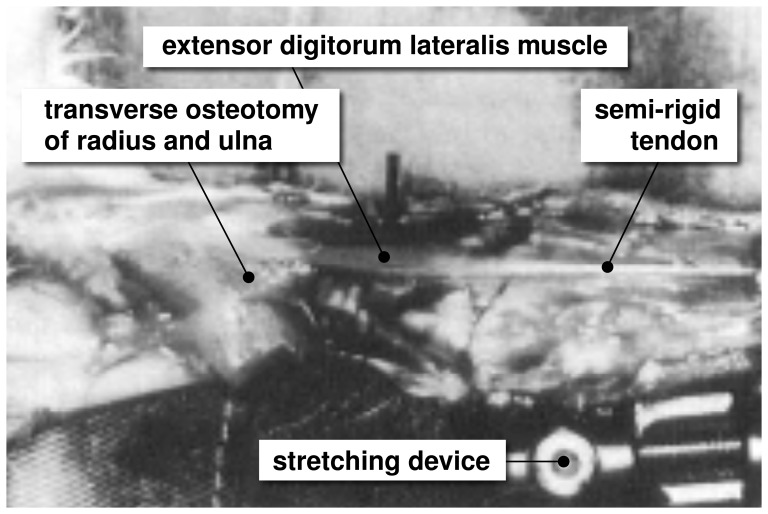
Stretching skeletal muscle. In a controlled limb lengthening model in rabbits, the radius and the ulna of the left forearm are lengthened by 4% inducing a stretch of 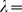
1.14 in the extensor digitorum lateralis muscle. Chronic eccentric muscle growth through sarcomerogenesis is characterized in situ using light diffraction imaging. Adopted with permission from [8].

Limb lengthening is a highly invasive surgical procedure to reconstruct or correct congenital and developmental deformities, post-traumatic injuries, regions of tumor removal, and short statue. Using the principle of distraction osteogenesis, the surgeon cuts the bone in two and gradually pulls apart the two ends, triggering new bone to form [5]. Figure 2 illustrates the procedure of limb lengthening through osteodistraction in the left forearm of an adult rabbit [8]. While the main goal of osteodistraction is to lengthen the bone itself, it is key to the procedure that the surrounding muscle grows in parallel with the stretched bone. Contracture, the lack of appropriate muscle adaptation, is a major source of complication during limb lengthening [9]. Optimal results can be obtained by lengthening the bone at a rate of 1 mm per day [5], up to no more than 20% of its initial length [9]. Although it is well accepted that mechanical factors play a limiting role in bone lengthening [10], to date, there are no mechanistic models that provide a scientific interpretation of these empirical guidelines.

In contrast to limb lengthening, tendon lengthening [11], tendon transfer [12], and tendon reattachment after tendon tear [13] are surgical procedures, which directly manipulate the muscle-tendon complex to correct posture or gait, and to improve or restore force generation. Typically, these corrections are performed in a single-step procedure, which permits the muscle to regain its original architecture [13]. Recent studies suggest that a restoration of normal architecture and physiological function might be possible through a gradual lengthening of the musculotendinous unit when stretched at a rate of 1 mm per day [14]. While we can sufficiently well approximate the short-term response to these surgical procedures by kinematic models [15], we are currently unable to predict their long-term behavior through chronic muscle adaptation.

In the clinical community, the dynamic adaptation of skeletal muscle is widely known as muscle plasticity [1]. This term suggests that the deformations caused by chronic lengthening are inelastic, i.e., they do neither store energy, nor do they generate stress [16]. In the mechanics community, mathematical models for growing of soft biological tissues indeed originate from finite strain plasticity [17]. They define growth through an incompatible configuration [18], characterized through the multiplicative decomposition of the overall deformation into a reversible elastic and an irreversible inelastic part [19], [20]. The irreversible part is represented through a second order growth tensor [21], which can be isotropic [22], transversely isotropic [23], [24], orthotropic [25], or generally anisotropic [26], [27] depending on the particular type of tissue. Its evolution can be driven by stress [28] or strain [29] again depending on the particular type of growth. Similar to cardiac muscle, skeletal muscle grows in a transversely isotropic pattern, along the fiber direction in response to passive mechanical stretch [25] and orthogonal to the fiber direction in response to active mechanical stress [30]. On the macroscopic scale, a passively stretched muscle grows eccentrically, i.e., it becomes longer, while an actively stressed muscle grows concentrically, i.e., it becomes thicker [31]. On the microscopic scale, passive stretch induces sarcomerogenesis, the serial deposition of sarcomere units, while active stress induces myofibrillogenesis, the parallel deposition of sarcomeres arranged in myofibrils [16], [32]. We have previously modeled both forms of growth in cardiac muscle [25] using generalized continuum theories with internal variables [21], [33]. However, to date, there is no mechanistic model to characterize growth in skeletal muscle using the nonlinear field theories of continuum mechanics.

Here we establish a mechanistic mathematical model for overstretch-induced eccentric skeletal muscle growth. The model is inherently multiscale, since it links macroscopic changes in elastic and inelastic muscle stretch to microscopic changes in sarcomere length and number. We illustrate the continuum theory of finite growth, adapt it to sarcomerogenesis, and embed it into a nonlinear finite element framework. We demonstrate that the model shows an excellent qualitative and quantitative agreement with experimentally measured sarcomere lengths and numbers in a chronic limb lengthening experiment.

## Methods

In this section, we briefly summarize the continuum modeling of sarcomerogenesis and its computational realization within a nonlinear finite element environment.

### Continuum Modeling of Sarcomerogenesis

To accurately represent the finite deformations during muscle stretching, we adopt the kinematics of finite growth, and introduce the deformation map 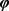, which, at any given time 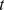, maps the material placement 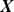 of a physical particle onto its spatial placement 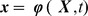. We then introduce the multiplicative decomposition of the deformation gradient [17],
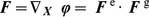
(1)into a reversible elastic part 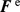 and an irreversible growth part 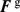 [19]. Here, 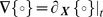 denotes the gradient of a field 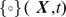 with respect to the material placement 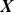 at fixed time 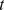. The Jacobian defines the overall change in tissue volume,
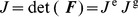
(2)which we can equivalently decompose into a reversibly elastic volume change 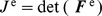 and an irreversibly grown volume change 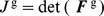. In muscle lengthening, growth is locally one dimensional, and the total muscle fiber stretch 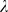 obeys a multiplicative decomposition similar to the deformation gradient itself [25]. We can interpret the total stretch 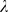 as a product of the reversible elastic stretch 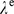 and the irreversible growth stretch 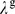,



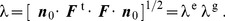
(3)We can then express the growth tensor 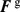 in terms of a single scalar-valued variable 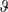, which represents the number of sarcomeres along the fiber direction 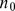 in the undeformed reference configuration [16], [21],
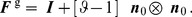
(4)


Since the muscle is not assumed to grow in the lateral direction, the serial sarcomere number 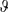 is not only identical to the irreversible growth stretch 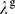, but also to the volume growth of the muscle, 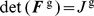,
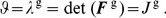
(5)


Using the simple rank-one update structure of 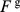, we can apply the Sherman-Morrison formula to invert the growth tensor, 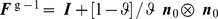, and obtain an explicit representation of the elastic tensor, 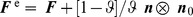. Here, 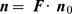 is the fiber direction in the deformed configuration. This allows us to explicitly introduce the elastic left Cauchy Green tensor.
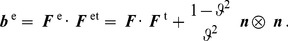
(6)


To focus on the impact of growth, we assume the passive muscle to behave isotropically elastic within the loading range of interest. We introduce the following Helmholtz free energy function.
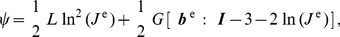
(7)where 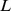 and 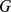 are the Lamé constants. We then evaluate the standard dissipation inequality to determine the Kirchhoff stress



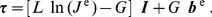
(8)This formulation implies that the newly created muscle will have the same microstructure, density, and stiffness, as the original, native tissue [14]. We model longitudinal muscle growth as a strain-driven process, and introduce the following evolution equation for the serial sarcomere number.
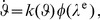
(9)in which 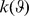 is a weighting function and 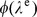 is a growth criterion similar to a yield function in the theory of plasticity. For the weighting function, we adopt a well-established functional form [34], which we rephrase here in a strain-driven format [16], [25] to control unbounded growth,



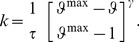
(10)The adaptation speed 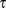 and the shape parameter for the adaptation curve 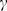 control the speed of adaptation [29], [35], and the maximum serial sarcomere number 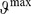 limits the maximum sarcomere deposition upon growth [24]. For the growth criterion, we assume that growth is driven by the elastic muscle fiber stretch 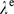. Guided by experimental observations [5], we activate growth only if the elastic fiber stretch exceeds a critical physiological limit 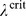,
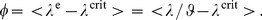
(11)


where 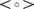 denote the Macaulay brackets.

### Computational modeling of sacromerogenesis

To solve the nonlinear finite element equations of stretch-induced muscle lengthening, we implement the growth model in a custom-designed version of the multipurpose nonlinear finite element program FEAP [36]. To characterize the growth process at each instant in time, we introduce the serial sarcomere number 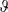 as an internal variable, and solve the biological equilibrium equation (9) locally at the integration point level [22], [37]. At each discrete time step 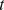, we determine the current serial sarcomere number 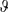 for a given current deformation state 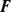 and a given area growth 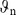 from the previous time step 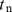 [16]. Accordingly, we introduce the following finite difference approximation for the material time derivative of the serial sarcomere number,
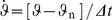
(12)where 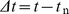 denotes the current time increment. In the spirit of implicit time stepping schemes, we now reformulate the evolution equation (9) with the help of equation (12) and introduce the discrete residual 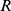 in terms of the unknown serial sarcomere number 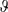.



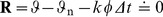
(13)We solve this nonlinear equation using a local Newton iteration [25]. Within each iteration step, we calculate the linearization of the residual 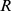 with respect to the serial sarcomere number 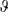,
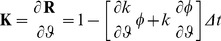
(14)in terms of the linearizations of the weighting function 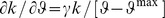 and the growth criterion 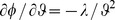 introduced in equations (10) and (11), see [23], [25]. We iteratively update the unknown serial sarcomere number,
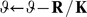
(15)until we achieve convergence, i.e., until the absolute value of the local growth update 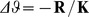 is below a user-defined threshold value. Once we have iteratively determined the current serial sarcomere number 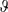, we can successively determine the growth tensor 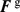 from equation (4), the elastic tensor 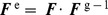, the Kirchhoff stress 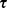 from equation (8), and, finally, the fourth order tensor 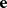 of the Eulerian constitutive moduli



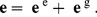
(16)The first term.
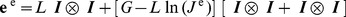
(17)


defines that standard elastic constitutive moduli where we have used the common abbreviations 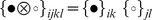 and 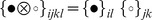, for the non-standard fourth order products. The second term.
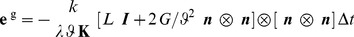
(18)depends directly on the constitutive formulation for the Kirchhoff stress 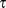 in equation (8), indirectly on the particular format of the growth tensor 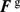 in equation (4), on the algorithmic linearization of the time discrete evolution equation for the serial sarcomere number 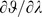 in equation (15), and on the linearization of the determinant 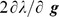 in equation (3). The local stress 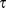 of equation (8) and the local consistent tangent moduli 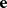 of equation (16) enter the global righthand side vector and the global iteration matrix of the global Newton iteration. Upon its convergence, we store the corresponding serial sarcomere number 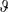 locally at the integration point level.

## Results

We illustrate the features of our model by means of three examples. In the first example, we calibrated the material parameter values of the growth model and compared the simulation against experimental findings of sarcomerogenesis in a chronic rabbit model. In the second and third examples, we analyzed temporal and regional variations of the microscopic sarcomere length and number, and explored their relations to the macroscopic elastic and inelastic stretch. Throughout all computational simulations, we used the same calibrated set of material parameters summarized in Table 1.

**Table 1:** Material parameters for elastic model and growth model.

	Interpretation	value	unit
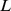	Lamé parameter	0.714	N/mm^2^
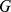	Lamé parameter	0.179	N/mm^2^
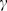	deposition nonlinearity	0.250	–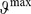	max serial sarcomere number	2.000	–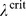	critical stretch threshold	1.000	–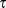	sarcomere deposition time	5.000	days

### Model Problem of Limb Lengthening: Experiment vs Simulation

To demonstrate the performance of the proposed model, we simulated the chronic stretching of the extensor digitorum lateralis muscle during limb lengthening, see Figure 2, and compared the simulation with experimental findings reported in the literature [8].

Experimentally, in a chronic limb lengthening model, the radius and the ulna of the left forearm of 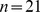 rabbits were lengthened by 3.5 mm through a transverse osteotomy, while 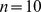 right forearms served as controls. This particular lengthening mimicked the difference between the length of the extensor digitorum lateralis muscle in full palmar wrist flexion and in full dorsiflexion. With an original ulnar length of 85 mm, the lengthening of 3.5 mm induced a bone stretch of 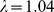. With an original original extensor digitorum lateralis length of 24 mm, the lengthening of 3.5 mm induced a muscle stretch of 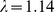. The study assumed that the connecting tendon was significantly stiffer than the stretched muscle, and therefore remained virtually unstretched [8]. Sarcomere lengths were recorded in the unstretched right forearm to serve as controls (n = 10), and in the stretched left forearm at days 0 (n = 5), 2 (n = 4), 5 (n = 4), 9 (n = 4), and 14 (n = 4) using light diffraction imaging.

Computationally, we modeled the stretched muscle as a homogeneous unit stretched by 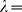
1.14. We restricted the maximum serial sarcomere number to 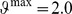 and chose the critical threshold for the onset of sarcomerogenesis to 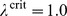. From a parameter calibration of the experimental measurements [8], we selected the sarcomere deposition time to 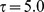 days and the sarcomere deposition nonlinearity to 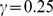. Using a time step size of 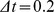 days, we simulated a chronic adaptation interval that exceeded the experimental interval of 14 days by an additional three days.

**Figure 3:**
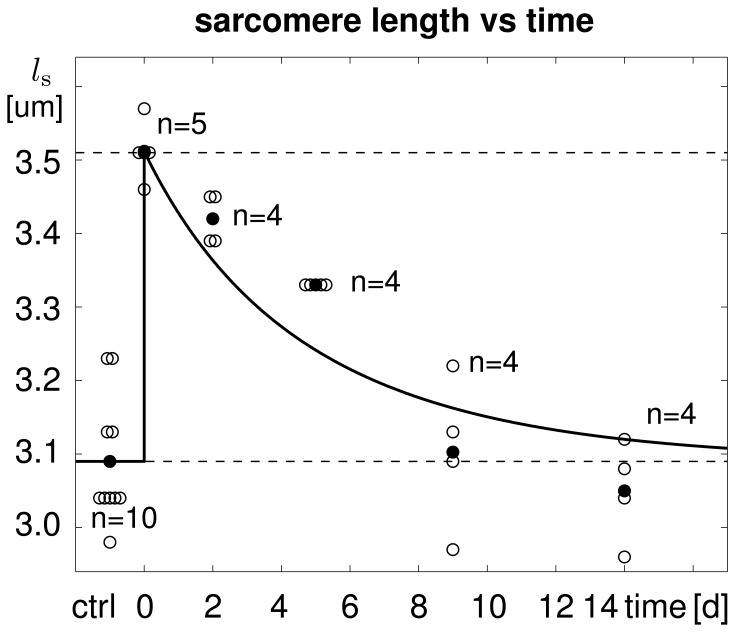
Temporal evolution of sarcomere length 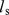 in chronically stretched skeletal muscle. Upon stretching the extensor digitorum lateralis muscle by 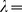
1.14, the sarcomere length increases acutely from 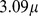
m to 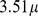
m and then returns chronically to its initial length of 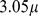
m within two weeks. Computationally predicted sarcomere lengths (solid line) agree nicely with experimentally measured sarcomere lengths (white circles) and their mean values (black circles) from [8].

Figure 3 illustrates the temporal evolution of the experimentally measured and computationally predicted sarcomere length 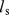, which increased acutely from 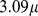
m to 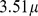
m and then returned chronically to its initial length of 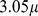
m within a period of two weeks. The adaptation process was clearly nonlinear, with a fast initial adaptation and a later convergence towards a steady state. Computationally predicted sarcomere lengths agreed nicely with their experimentally measured counterparts.

**Figure 4:**
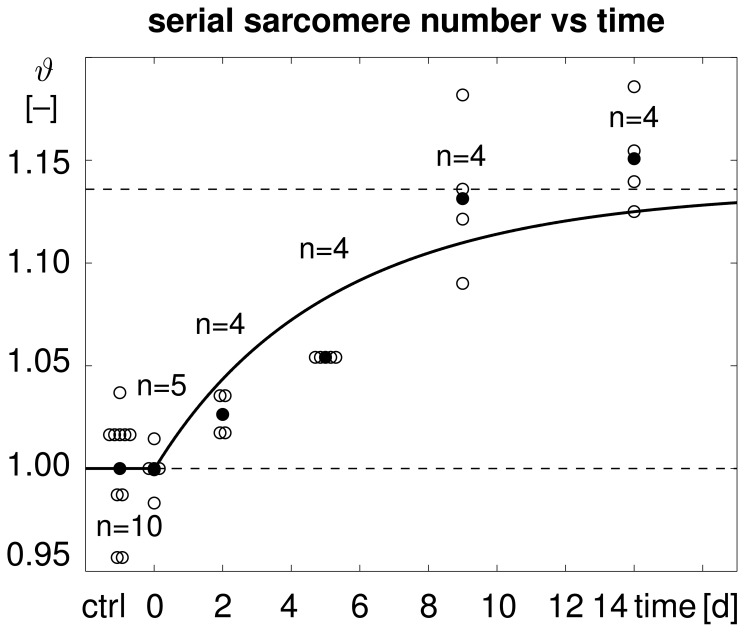
Temporal evolution of serial sarcomere number 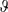 in chronically stretched skeletal muscle. Upon stretching the extensor digitorum lateralis muscle by 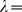
1.14, the sarcomere number increases gradually from 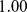 to 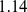 within two weeks, bringing the sarcomere length 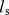 back to its initial value. Computationally predicted sarcomere numbers (solid line) agree nicely with experimentally measured sarcomere numbers (white circles) and their mean values (black circles) from [8].

Figure 4 illustrates the temporal evolution of the experimentally measured and computationally predicted serial sarcomere number 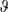, which increased gradually from 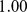 to 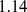 within two weeks. This reduced the elastic stretch and brought the sarcomere length back to its initial value. Computationally predicted sarcomere numbers agreed nicely with their experimentally measured counterparts.

Table 2 summarizes the experimentally measured and computationally simulated sarcomere lengths, along with the corresponding experimental standard deviations and simulation errors. The relative standard acute error of the computational prediction, i.e., the acute error at day 0, was 0.00% by model design. The average relative standard chronic error of the computational prediction, i.e., the average chronic error at days 2, 5, 9, and 14, was 2.13%. At all time points, the simulation error was of the same order of magnitude as the experimental standard deviation.

**Table 2:** Sarcomere lengths in chronically stretched skeletal muscle.

	ctrl	d0	d2	d5	d9	d14
experiment [ 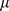 m]	3.09	3.51	3.42	3.33	3.10	3.05
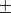 std [ 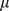 m]	0.09	0.04	0.03	0.00	0.10	0.07
simulation [ 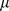 m]	3.09	3.51	3.36	3.24	3.16	3.12
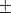 error [ 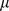 m]	0.00	0.00	0.06	0.09	0.06	0.07
error [% ]	0.00	0.00	1.65	2.66	1.93	2.30

Computationally predicted sarcomere lengths agree nicely with experimentally measured sarcomere lengths with errors on the order of the experimental standard deviation [8].

### Model Problem of Limb Lengthening: Single-step vs Multi-step Stretching

To illustrate a potential application of the proposed model in therapeutic protocol design, we simulated and compared the chronic stretching of the extensor digitorum lateralis muscle during a single-step and a multi-step limb lengthening procedure.

**Figure 5:**
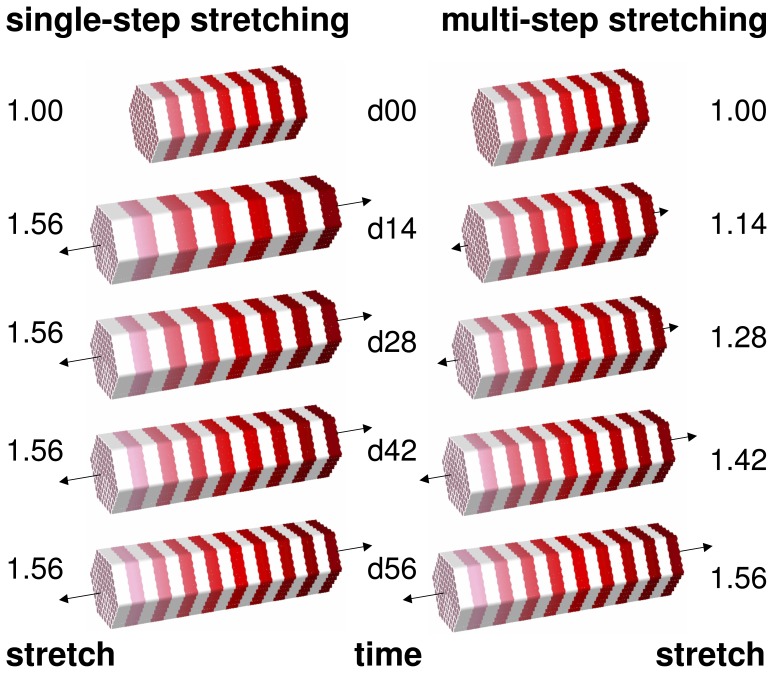
Single-step stretching vs multi-step stretching of skeletal muscle. A total stretch of 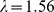 is applied ad hoc (left) and gradually (right). Sarcomerogenesis is simulated for over period of eight weeks.

Figure 5 illustrates the simulated procedure with the total stretch applied in a single step of 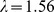, shown on the left, and applied in four steps of 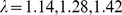 and 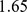, shown on the right. We utilized the material parameters identified in the previous section, i.e, a maximum serial sarcomere number of 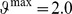, a critical threshold for the onset of sarcomerogenesis of 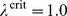, a sarcomere deposition time of 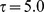 days, a sarcomere deposition nonlinearity of 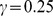, and a time step size of 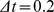 days. This implied that the first step of the multi-step simulation was identical to the simulation of the previous section, see Figure 5, top right.

**Figure 6:**
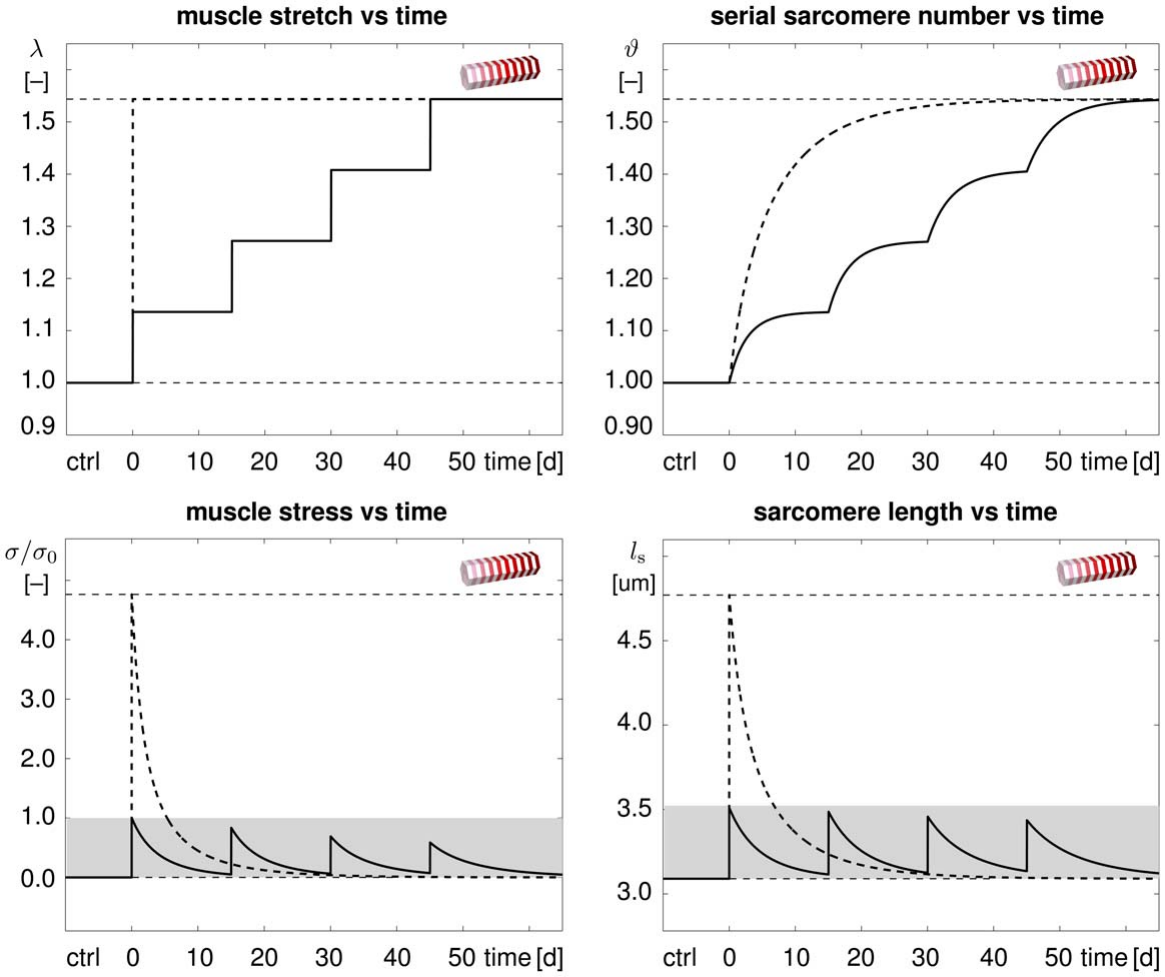
Temporal evolution of muscle stretch 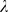, muscle stress 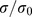, serial sarcomere number 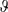, and sarcomere length 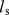 in chronically stretched skeletal muscle. Single-step stretching (dashed lines) induces a drastic change in stretch resulting in a pronounced overstress and sarcomere lengthening. Multiple-step stretching (solid lines) induces a gradual change in stretch inducing a moderate overstress and sarcomere lengthening. Muscle stress and sarcomere length stay within their physiological regimes (gray box).

Figure 6 illustrates the temporal evolution of the macroscopic quantities, muscle stretch and muscle stress, and of the microscopic quantities, serial sarcomere number and sarcomere length. The single-step stretching procedure indicated through the dashed lines induced a drastic change in stretch, top left, resulting in a pronounced overstress, bottom left, and pronounced sarcomere lengthening, bottom right. The multi-step stretching procedure indicated through the solid lines induced a gradual change in stretch, top left, resulting in a moderate overstress, bottom left, and moderate sarcomere lengthening, bottom right. While single-step stretching induced unphysiologically large muscle stresses and sarcomere lengths, multi-step stretching kept both these values within their physiological regimes, indicated through the gray boxes.

### Clinical Problem of Biceps Tendon Tear

To illustrate the potential of the proposed model in stretch-induced lengthening of a realistic muscle geometry, we simulated sarcomerogenesis in the biceps brachii muscle. Stretch-induced re-lengthening might become necessary after complete tendon tear, when the retracted distal or proximal biceps tendon is surgically reattached to the bone [13].

**Figure 7:**
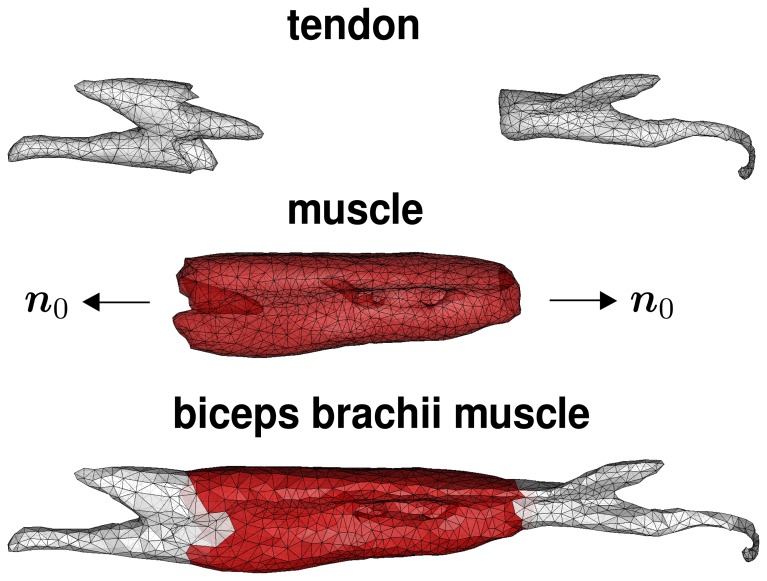
Biceps brachii muscle. The finite element model reconstructed from magnetic resonance images consists of 2,705 nodes and a total of 11,816 linear tetrahedral elements. The muscle tissue, discretized by 9,393 elements (red), is attached to the elbow (left) and to the shoulder (right) through tendon tissue, discretized by 2,423 elements (gray). The biceps brachii is a classical fusiform muscle with fibers 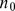 arranged in parallel bundles along its long axis [38].

Figure 7 illustrates our finite element model of the biceps brachii muscle, reconstructed from magnetic resonance images [38], [39]. We discretized the muscle-tendon unit with 11,816 linear tetrahedral elements connected at 2,705 nodes. The muscle consisted of 9,393 elements, shown in red. The distal and proximal biceps tendons, which connect the muscle to the elbow, left, and to the shoulder, right, consisted of 2,423 elements, shown in gray. Since the biceps brachii is a classical fusiform muscle [38], we assumed that its fibers are arranged in parallel bundles along its long axis indicated through the vector 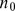.

We modeled the reattachment of the tendon after tendon tear, by lengthening the 40 cm long muscle-tendon unit by 2 cm. Here we were not particularly interested in the forces needed to apply this particular stretch [13]. Accordingly, for simplicity, we selected a simple Neo-Hookean elastic model with 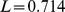
N/mm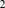 and 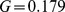
N/mm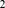 for the muscle tissue. Since the tendon tissue is more than one order of magnitude stiffer than the muscle tissue [40], we modeled the distal and proximal tendons as semi-rigid. For the muscle tissue, we adapted the model parameters calibrated in the previous section, and chose the maximum serial sarcomere number to 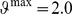, the critical threshold for the onset of sarcomerogenesis to 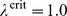, the sarcomere deposition time to 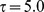 days, and the sarcomere deposition nonlinearity to 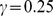. We used a time step size of 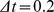 days and simulated a chronic adaptation interval of 14 days.

**Figure 8:**
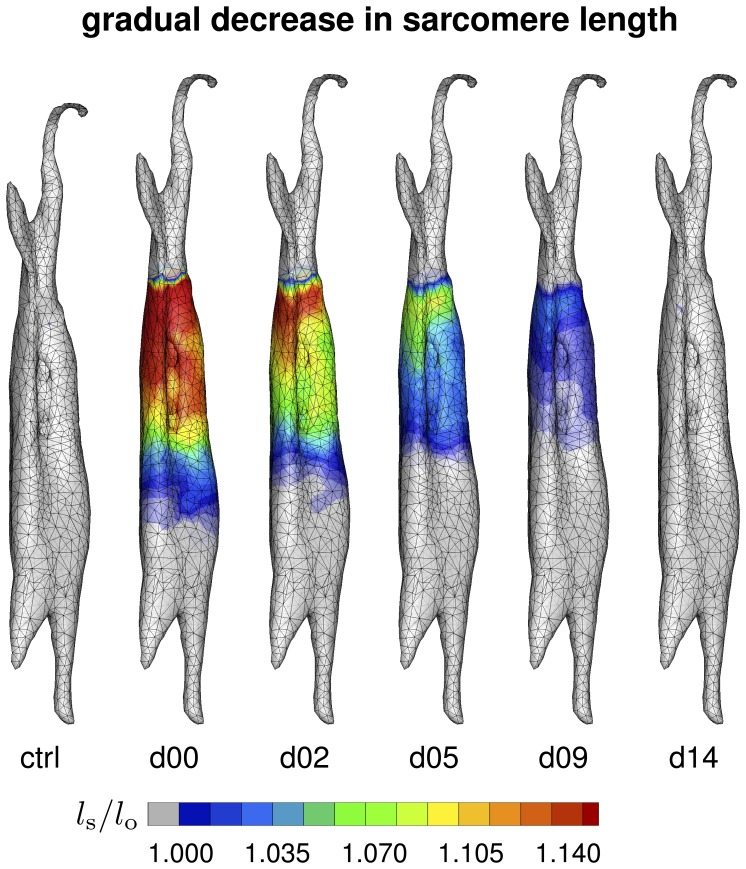
Spatio-temporal evolution of sarcomere length 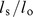 in chronically stretched skeletal muscle. Upon lengthening the biceps brachii muscle by 2 cm, i.e., by 5%, the sarcomere length increases acutely from 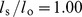 to 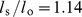 and beyond, and then returns chronically to its initial value of 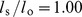 within two weeks. The sarcomere length 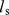 is a measure for the elastic fiber stretch 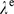.

Figure 8 illustrates the spatio-temporal evolution of the sarcomere length 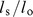 in the control state, left, and in the stretched state at day 0, day 2, day 5, day 9, and day 14, right. A total lengthening of 2 cm stretched the overall muscle unit by 5% along its long axis. Since the tendon tissue was assumed to be semi-rigid, this resulted in an average sarcomere length of approximately 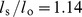. Upon stretching, the sarcomere length increased acutely from 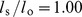 to 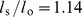 on day 0, and then returned chronically to its initial value of 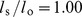 within the simulated period of two weeks. Figure 8 demonstrates that the sarcomere length displayed a significant regional variation. Proximally, at the shoulder side, where the muscle-tendon interface is relatively sharp, the sarcomere length was 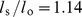 and larger, see Figure 8, top. Distally, at the elbow side, where the stiff tendon branches into the soft muscle tissue, the sarcomere length was 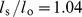 and smaller, see Figure 8, bottom. The sarcomere length 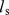 is a measure for the elastic fiber stretch 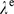.

**Figure 9:**
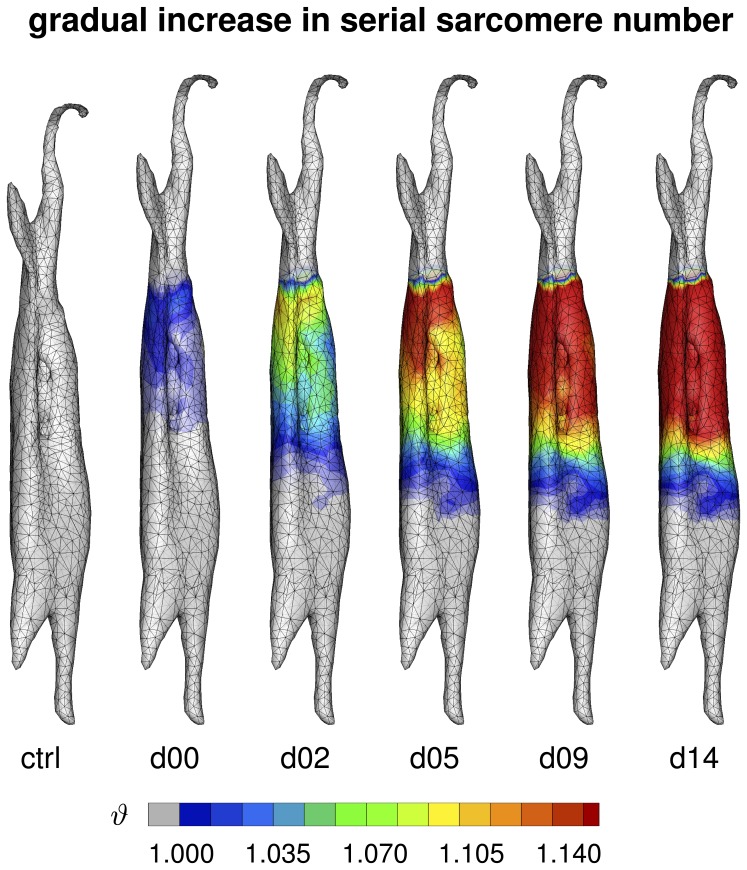
Spatio-temporal evolution of serial sarcomere number 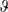 in chronically stretched skeletal muscle. Upon stretching the biceps brachii muscle by 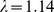, the serial sarcomere number increases gradually from 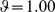 to 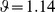 within two weeks, brining the sarcomere length back to its initial value of 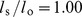. The serial sarcomere number 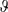 is a measure for the inelastic fiber stretch 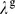.

Figure 9 illustrates the spatio-temporal evolution of the serial sarcomere number 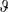 in the control state, left, and in the stretched state at day 0, day 2, day 5, day 9, and day 14, right. Upon stretching the biceps brachii muscle by 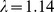, the serial sarcomere number increased gradually from 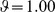 to 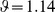 within two weeks, while, at the same time, the sarcomere length decreased from 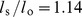 to 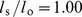. Similar to the sarcomere length, the serial sarcomere number displayed a significant regional variation, with largest values and sharp profiles proximally, at the shoulder side, and smallest values and smooth profiles distally, at the elbow side. The serial sarcomere number 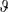 is a measure for the inelastic fiber stretch 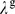.

**Figure 10:**
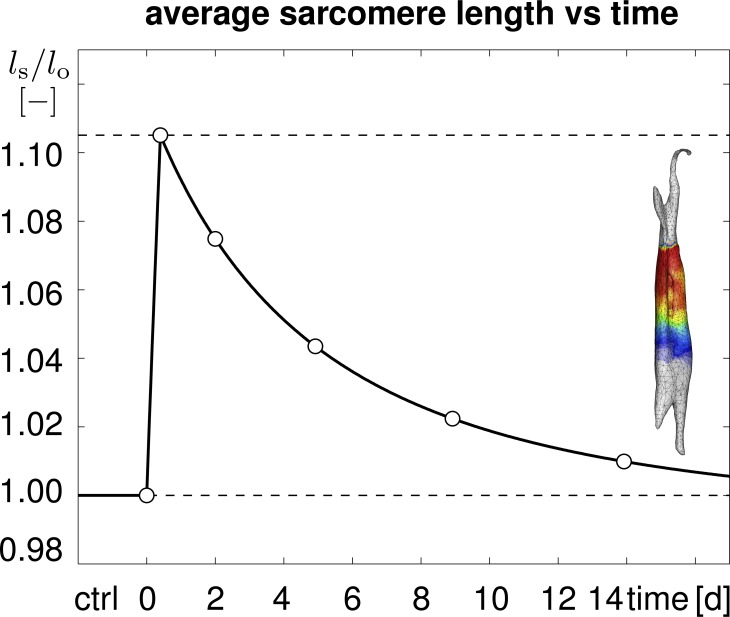
Temporal evolution of average sarcomere length 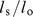 in chronically stretched skeletal muscle. Upon stretching the biceps brachii muscle by 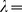
1.14, the average sarcomere length increases acutely to 1.105 times its initial length and then returns chronically to its initial length within two weeks. Averaged sarcomere length at discrete time points (white circles) correspond to the volume averaged elastic stretches 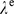, averaged over the muscle tissue region in Figure 8.

Figure 10 displays the temporal evolution of the average sarcomere length 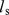 calculated as the volume average of the elastic stretch 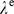, scaled by the initial sarcomere length 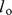. The average sarcomere length increased rapidly by almost 0.105, and then decreased back to its initial length within a period of 14 days. Qualitatively, the shape of the curve corresponds to the evolution of the sarcomere length in the limb lengthening experiment of Figure 3. Quantitatively, the averaged sarcomere length at days 0, 2, 5, 9, and 14, indicated through the white circles, correspond to the volume averaged elastic stretches 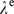 illustrated in Figure 8.

**Figure 11:**
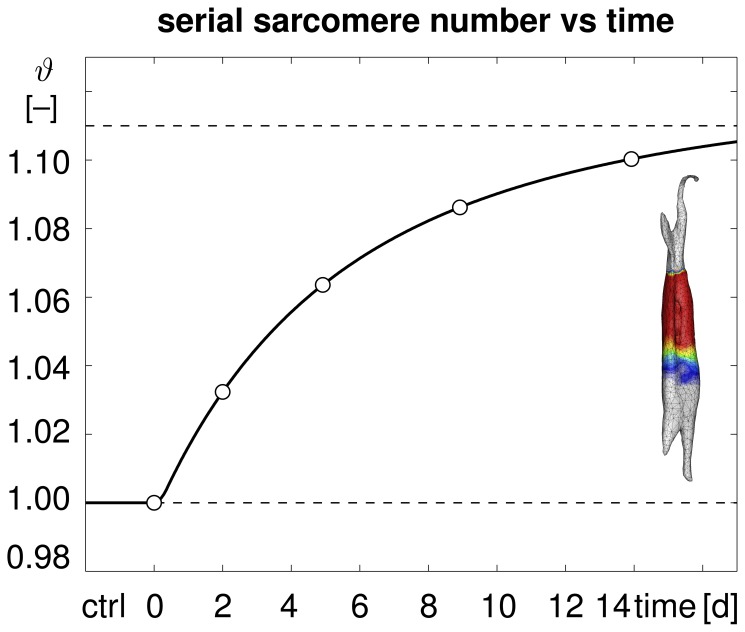
Temporal evolution of serial sarcomere number 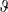 in chronically stretched skeletal muscle. Upon stretching the biceps brachii muscle by 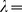
1.14, the sarcomere number increases gradually from 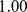 to 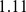 within two weeks, bringing the individual sarcomere lengths 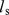 back to their initial values. Sarcomere numbers at discrete time points (white circles) correspond to the volume averaged inelastic stretches 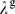, averaged over the muscle tissue region in Figure 9.

Figure 11 displays the temporal evolution of the total sarcomere number 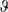 scaled by the initial sarcomere number. The average sarcomere number increased smoothly by 0.105 within a period of 14 days. Qualitatively, the shape of the curve corresponds to the evolution of the sarcomere number in the limb lengthening experiment of Figure 4. Quantitatively, the sarcomere numbers at days 0, 2, 5, 9, and 14, indicated through the white circles, correspond to the volume averaged inelastic stretches 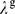 illustrated in Figure 9.

## Discussion

We have proposed, for the first time, a mechanistic multiscale model for stretch-induced sarcomerogenesis, in which chronic muscle lengthening is characterized through a scalar-valued internal variable, the serial sarcomere number. The model interprets the macroscopic elastic and inelastic fiber stretches 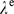 and 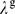 as metrics for the microscopic sarcomere length and sarcomere number 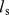 and 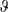. It is in excellent qualitative and quantitative agreement with the sarcomere lengths observed in a chronic limb lengthening experiment.

### Limitations

Although our first prototype model agrees nicely with experimental findings, a few limitations remain to be addressed in future model refinements. First, for the sake of simplicity, we have chosen a relatively straightforward baseline elastic model, see equation (7). Since we model the growth process as strain-driven [23] and not stress-driven [22], the choice of the constitutive model affects the growth process only indirectly. An appropriate muscle model would become important though if we wanted to predict limit stresses and forces required to apply the desired stretch [13]. However, as our approach is inherently modular, it would be relatively straightforward to integrate a more physiological constitutive model [41], [42]. Second, since tendon tissue is more than one order of magnitude stiffer than muscle tissue [38], [40], we have modeled the tendon as semi-rigid. This approach provides quick insight into overall characteristics and trends. Extending the model towards a tendon with a finite stiffness should not pose additional complexity, provided the tendon model parameters are known [40]. Third, we have assumed that muscle lengthening translates directly into sarcomere lengthening, i.e., that the elastic fiber stretch is directly correlated to the sarcomere length [43]. This approach is relatively common in the skeletal muscle literature [8], [15], although potential second order effects could possibly contribute to additional muscle lengthening. Fourth, we have adopted a simple functional form for the evolution of the sarcomere number in equations (9) and (10). This particular format is conceptually well-understood since it has been applied to model growth of other soft biological tissues, first in a stress-driven [28], [34] then in a strain-driven [25], [30] version. Although this format seems to yield an excellent agreement with experimental findings, alternative evolution equations might be possible and could be integrated in a relatively straightforward fashion. Along the same lines, we could further enhance the model to integrate sarcomere disassembly upon chronically reduced stretch [12], a phenomenon that has been studied intensely during immobilization [7], [44] and tendon retraction [13]. Last, to better calibrate the model, a richer data set would be desirable. Here we have based our model calibration on a two-week long limb lengthening study in rabbits [8]. At this point, it is unclear whether the adaptation speed observed in small animals translates directly to humans. Currently, the lack of chronic experiments with multiple well-defined time points still limits the clinical use of the model. However, recent developments in second harmonic generation microendoscopy [45] now allow us to measure local sarcomere length non-invasively in humans, to precisely quantify spatial and temporal sarcomere variations in vivo.

### Significance

Our model is the first mechanistic model to link macroscopic elastic and inelastic stretch to microscopic sarcomere length and number using nonlinear continuum theories of finite growth. On the microscopic scale, chronic muscle stretching beyond the physiological limit creates unphysiologically large sarcomere lengths [46], which, in turn, induce a serial sarcomere deposition [5]. The resulting increase in sarcomere number causes a chronic restoration of the initial sarcomere length [6]. On the macroscopic scale, the serial sarcomere deposition induces a chronic reduction of the macroscopic elastic stretch, gradually reducing the passive stress [16], a phenomenon similar to classical stress relaxation [47]. Our model provides a mechanistic understanding of the underlying mechanisms accompanying chronic muscle stretch and sarcomerogenesis [24]. At this point, it does not describe the mechanobiology and the mechanotransduction pathways associated with sarcomerogenesis [3]. However, ultimately, it would be desirable to tie the mechanical response to mechanoreception, intracellular signaling pathways, and target activation [10].

We have shown that our model can explain why gradual multi-step stretching is less invasive than single-step stretching [9]. It also explains regional variation in sarcomere lengths [48], shorter close to and longer away from the muscle-tendon interface, where the stiff tendon provides additional support to stretch [43]. Acutely, our model could serve as a design tool to prevent short-term muscle damage caused by mechanical overstretch. Macroscopically for a given limit stress, or equivalently, microscopically for a given limit sarcomere length, we could optimize temporal stretching sequences that predict the maximum possible stretch within acceptable limits [14]. The ultimate goal would be to maximize stretch-induced muscle growth, such that the muscle always stays within a physiologically reasonable operating range. Chronically, our model could serve as a design tool to predict long-term muscle adaptation. We could easily integrate it into existing skeletal muscle models to optimize muscle lengthening in response to eccentric training [49] or to predict different surgical procedures such as tendon transfer, tendon reattachment, or tendon lengthening [11]. The ultimate goal would be to guarantee optimal regeneration and long-term repair durability.

### Conclusion

Striated muscle adapts to chronic mechanical stretch through the creation and serial deposition of new sarcomere units. The phenomenon of sarcomerogenesis has been quantified in chronic animal experiments, but it has never been modeled computationally. Here we have presented a mathematical model for chronic muscle growth through sarcomerogenesis and illustrated its computational realization. Acutely, upon 14% lengthening of the extensor digitorum lateralis muscle, the model predicts a sarcomere lengthening from 3.09
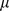
m to 3.51
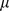
m with a model error of 0.00%. Chronically, the model predicts the gradual return to the initial sarcomere length within two weeks with a model error of 2.13%, which is within the rage of the experimental standard deviation. Once calibrated with a richer data set, our model may be used to help surgeons to make informed decisions about optimal stretch increments, stretch timing, and stretch amplitudes. Our study might to open new avenues in orthopedic and reconstructive surgery and enhance muscle adaptation, repair, and regeneration for patients with ill proportioned limbs, tendon lengthening, tendon transfer, tendon tear, or chronically retracted muscles.
